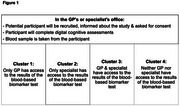# The Community General Practitioner and General Specialist‐based Cognitive Screening to Identify Early Decline in Seniors in Germany Study (COGSCREEN 2)

**DOI:** 10.1002/alz70857_099980

**Published:** 2025-12-24

**Authors:** Robert Perneczky, Anna Hufnagel, Carolin Isabella Kurz, Paulina Tegethoff

**Affiliations:** ^1^ German Center for Neurodegenerative Diseases (DZNE), Munich, Germany; ^2^ Munich Cluster for Systems Neurology (SyNergy), Munich, Germany; ^3^ Ageing Epidemiology (AGE) Research Unit, School of Public Health, Imperial College London, London, United Kingdom; ^4^ Sheffield Institute for Translational Neuroscience, University of Sheffield, Sheffield, United Kingdom; ^5^ Department of Psychiatry and Psychotherapy, University Hospital, LMU Munich, Munich, Germany; ^6^ Department of Psychiatry and Psychotherapy, LMU Hospital, LMU Munich, Munich, Germany

## Abstract

**Background:**

While knowledge about dementia and its causes is increasing rapidly, healthcare systems remain ill‐equipped to detect cognitive decline in the early stages of neurodegenerative diseases such as Alzheimer's disease (AD). However, improving the early identification of AD in the population is a prerequisite for dementia prevention and providing future disease‐modifying treatments for individuals most likely to benefit. Subjective cognitive deficits (SCD) and mild cognitive impairment (MCI) may indicate prodromal AD, even in the absence of functional impairment; in conjunction with an AD‐typical biomarker profile, the risk of further cognitive decline increases significantly. Offering cognitive and biomarker investigations to individuals with SCD or MCI may therefore open a window of opportunity for early interventions.

**Method:**

This project, part of the Davos Alzheimer's Collaborative Healthcare System Preparedness Accurate Diagnosis Project, builds on a network of general practitioners (GPs) and specialists in private practice (neurologists, psychiatrist and geriatricians) in Munich, Germany. We will introduce participating physicians to a proprietary digital cognitive test (developed by Medotrax) and blood‐based biomarkers (Roche *p*‐tau217). GP‐specialist pairs will be allocated to four groups of centers, with varying access to biomarker tests (Figure 1). The main aim of the study is to assess the percentage of AD diagnoses made with biomarker evidence, with secondary aims including the impact of blood‐based and digital investigations on resource utilization and diagnostic workflows.

**Result:**

Initial experiences with designing the study protocol, learnings from the recruitment of study sites and insights from study participants will be presented.

**Conclusion:**

Currently, there is no system in place for targeted, standardized identification of cases with minimal cognitive decline in Germany or worldwide, hindering efforts to detect neurodegenerative and other causes of cognitive impairment in large segments of the population. The lack of a robust approach for detecting early changes with acceptable accuracy outside of specialist clinics results in disappointingly low diagnostic rates. The COGSCREEN 2 study will help to establish an effective and efficient early diagnosis framework, embedded in a global network of DAC sites.